# Experimental Study on Effects of Additional Prestressing Using Fiber Reinforced Polymers and Strands on Deterioration of PSC Bridge Structure

**DOI:** 10.3390/polym14061115

**Published:** 2022-03-10

**Authors:** Tae-Kyun Kim, Woo-Tai Jung, Jong-Sup Park, Hee-Beom Park

**Affiliations:** 1 Department of Structural Engineering Research, Korean Institute of Civil Engineering and Building Technology, 283, Goyang-daero, Ilsanseo-gu, Goyang-si 10223, Gyeonggi-do, Korea; woody@kict.re.kr (W.-T.J.); jspark1@kict.re.kr (J.-S.P.); 2 Department of Highway & Transportation Research, Korean Institute of Civil Engineering and Building Technology, 283, Goyang-daero, Ilsanseo-gu, Goyang-si 10223, Gyeonggi-do, Korea; heebeompark@kict.re.kr

**Keywords:** deteriorated bridge, addition prestressing, fiber-reinforced polymer, strengthening method, external prestressing, near-surface mounted, prestressed concrete

## Abstract

Concrete bridge structures require reinforcement, as their performance deteriorates over time. In this regard, this study evaluated the effect of additional prestressing using fiber-reinforced polymers (FRPs) and strands applied to a demolished, deteriorated bridge. In particular, specimens were prepared for a bridge subjected to non-, near-surface mounted (NSM), and external prestressing (EP) strengthening to evaluate the stiffness and safety of the structure. In the 200–400 kN load range, the EP method exhibited the highest stiffness (15 kN/mm), followed by non-strengthening (8.5 kN/mm) and the NSM method (5.45 kN/mm). The EP method increased the stiffness by approximately two times; however, the NSM method decreased the stiffness by 0.6 times. In the 400–800 kN load range, the EP and NSM methods yielded stiffness values of 2.58 and 0.7 kN/mm, respectively. These results confirm that the EP method reinforces the structure. The results of this study are expected to be used as basic data to reinforce deteriorated bridges in actual operation.

## 1. Introduction

Among reinforced concrete (RC) and prestressed concrete (PSC) structures, bridge structures have recently been exhibiting rapid performance degradation in terms of their usability and stability owing to the corrosion of steel [1,2]. The number of deteriorated bridges has also been increasing. Consequently, from an economic perspective, the corresponding maintenance costs have increased [3,4]. In South Korea, the current number of PSC bridges aged 30 years and over is 134, which is expected to increase to 369 over the next decade [5]. In addition, the total number of RC bridges aged 30 years and over is 273, which is also expected to increase considerably to 847 over the next decade [5]. Currently, these deteriorated bridges are collapsing due to various causes; more importantly, the collapse of large structures tends to directly lead to human casualties and property damage [1,2,5]. There are numerous examples of such bridge collapse accidents worldwide [6,7,8]. For instance, in the United States, the Tacoma Narrows, Silver, Sunshine Skyway, Mianus River, Schoharie Creek, Lowe Motor’s Speedway Pedestrian, and I-35W bridges collapsed due to natural disasters, repeated external loads (fatigue load), and steel corrosion, resulting in hundreds of human casualties and considerable property damages [6]. Furthermore, in Japan, bridges collapsed in the Nagano and Gifu Prefectures due to the corrosion of PS tendons and PC steel [6]. In Korea, collapse accidents also occurred at the Seongsu and International Bridges. In this regard, the performance of most structures deteriorates abruptly over time. In particular, problematic and collapse phenomena occur within 20 to 30 years [7,8]. Therefore, periodic safety diagnoses and inspections are required, and reinforcement must be performed when these problems arise. Reinforcement is performed to restore or improve the mechanical performance of members or structures, such as their load-carrying capacity and stiffness [9].

The most suitable strengthening method must be applied in the cases of deteriorated bridges, according to the site and structure conditions. Given that the performance of internal steel materials deteriorates over time in deteriorated bridges, an additional prestressing method can be applied for reinforcement to increase the stiffness of members, and the material substitution method can be adopted to prevent the corrosion of steel [9]. Among the various existing strengthening methods, the external prestressing (EP) method enabling additional prestressing [10,11,12,13,14] and the near-surface mounted (NSM) method [15,16,17] have been used. Under the NSM method, steel is replaced with fiber-reinforced polymers (FRPs) exhibiting a high-tensile strength, low weight, and corrosion resistance. By contrast, the EP method entails the use of strands. In both these methods, additional prestressing is applied upon completing construction. With regard to FRPs, glass fiber-reinforced polymers, such as aramid FRPs (AFRPs) and carbon FRPs (CFRPs) have been used as major strengthening materials [18]. FRPs exhibit brittle failure but have superior strength, weight, durability, creep, and fatigue resistance, compared to existing construction materials [19,20,21,22]. In addition, their easy handling in the field owing to their low weight, the possibility of rapid installation with a small number of people, and their high-corrosion resistance have expanded the applicability of FRPs [23]. In particular, carbon fiber has a significantly lower density, high-tensile strength, high-modulus of elasticity, low weight, low thermal expansion coefficient, and superior strength (up to 10 times), compared to iron, although its weight is ¼ that of iron. Recently, this material has garnered considerable interest as a lightweight material for the automobile, construction, and sports industries. However, the use of this material is limited by its high price. Notably, several previous studies have focused on various strengthening methods. Zhang et al. [24] applied the NSM method to a 3 m model beam and evaluated its structural performance according to the load point. Jahani et al. [25] applied the NSM method to a 2.4 m beam according to the number of CFRPs and evaluated its structural performance with respect to temperature. Abdallah et al. [26] applied the NSM method to a 3 m beam and evaluated the performance of an RC structure according to the reinforcement height. Le et al. [27] fabricated a T-shaped RC structure with a length of 3 m, applied the EP method using reinforcing bars and CFRPs, and compared the performance. Zou et al. [28] used a 3 m T-beam RC structure and evaluated its performance according to the reinforcement arrangement under the EP method [24,25,26,27,28]. Furthermore, Guo et al. [29] evaluated the effect of the type of fiber hybridization on the mechanical properties of carbon/glass FRPs. Li et al. [30] proposed a novel wedge-shaped bond anchorage system for pultruded CFRP plates. Lastly, Sun et al. [31] proposed a novel strong and durable NSM FRP method with cost-effective fillers. However, as described above, previous studies mainly employed models or small-scale structures for most strengthening methods. In contrast to previous studies, in this study, strengthening methods were applied to real-scale structures used for decades, and their performances were evaluated. The results thus obtained can serve as a database to induce ductility destruction of the behavior of the FRP reinforcement with unclear yield points in the reinforcement design in the future.

In this study, additional prestressing using FRPs and strands was applied to a demolished deteriorated bridge. In particular, specimens were prepared based on a bridge subjected to non-, NSM, and EP strengthening to evaluate the stiffness and safety of the structure. After reinforcement, the performance of the structures should be higher than that before reinforcement. In the 200–400 kN load range, the EP method exhibited the highest stiffness (15 kN/mm), followed by non-strengthening (8.5 kN/mm) and the NSM method (5.45 kN/mm). The EP method increased the stiffness by approximately two times, but the NSM method decreased the stiffness by 0.6 times. These strengthening methods were previously studied mainly based on models or small-scale structures. However, in this study, these methods were applied to large, deteriorated structures that were actually demolished. The results of this study could be used to develop a database that can be used for reinforcements at construction sites in the future.

## 2. Materials and Methods

### 2.1. Strengthening Methods

The NSM method used in this study involves external bonding (EB). Its basic principle is to combine reinforcement with a concrete structure using an adhesive to achieve the reinforcement effect. Therefore, accurate information regarding the properties of the reinforcement is required, and the interface between concrete and the reinforcement, i.e., the adhesive layer, must exhibit reliable adhesion performance, while sufficiently securing durability. Strands are fabricated by twisting two or more wires. In general, seven-wire strands are most frequently used in structures. For seven-wire strands, six wires are arranged around the core wire, i.e., the wire in the center and these are referred to as lateral wires. In this study, seven-wire strands were used as strands, and four 15.2 mm wires were arranged accordingly. Note, that losses may occur when prestressing is applied to a structure; these losses can be classified into short- and long-term losses. Short-term loss involves the occurrence of anchorage slip, elastic deformation, and friction, whereas long-term loss involves the occurrence of creep, relaxation, and shrinkage.

### 2.2. Specification of Specimens

**Figure 1:**
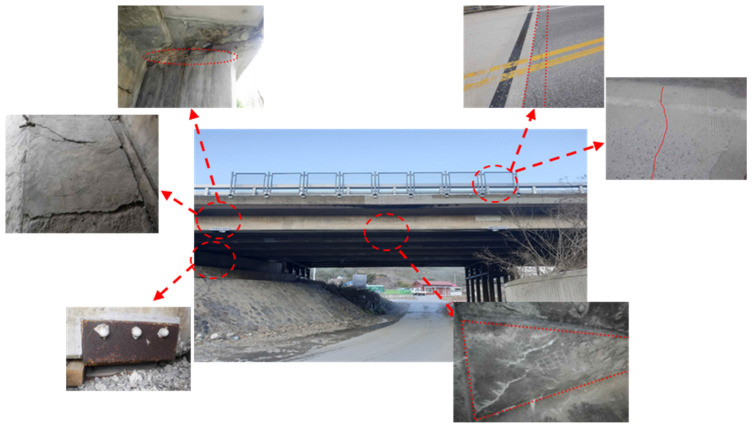
Bridge before demolition.

The specimens used in this study were based on an actual bridge (OO Bridge) located in Gangneung, South Korea. The bridge, shown in Figure 1, was used for 43 years as it was completed in 1977. The structure is a PSC-I type bridge with a span of 25.8 m and a width of 18.5 m. The bridge was previously reinforced with fibers for the deck and with EP for the external girders. The bridge grade is DB-18, and the condition grade is D. The bridge was demolished on 10 March 2019. As it was considerably old, finding design data or confirming its basic specifications was challenging owing to the resolution of the drawings. Therefore, the specifications of the bridge were identified based on actual measurements or basic property tests. In particular, 12 tendons were inserted into each of the six ducts. In the case of actual measurements, the span bridges used for the NSM and EP methods were measured and found to be similar.

**Figure 2:**
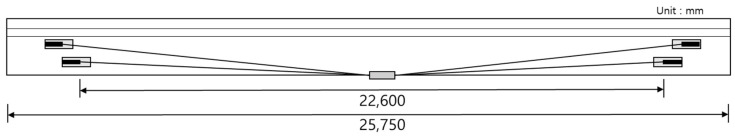
Geometry of specimen reinforced with the external prestressing (EP) method.

Figure 2 shows the specimen configuration employed to study the EP method. The total length is 25,750 mm, and the reinforcement length is 22,600 mm. EP is a method of applying prestressing to the outside of the structure, without placing tendons inside the structure. Unlike the internal prestressing method, where the sheath is filled with grout between the tendons and the structure after prestressing, the tendons generally remain unbonded and do not exhibit an integrated behavior with the target structure in the EP structure-strengthening method. This approach is applied to increase the load-carrying capacity of existing bridges subject to environmental changes or to restore the load-carrying capacity of deteriorated bridges. This method introduces prestressing by installing anchorage devices and saddles in the existing concrete members and PS tendons outside the members. The method is used for strengthening the entire structure rather than locally repairing the structure. Furthermore, this method is typically applied to improve the stress state in concrete members, crack control, and deflection or to change the structural system, such as the continuity in simple bridges. As deviators (saddles) are generally installed on the outer parts of the girders in the EP method, the method differs from the internal prestressing method, where deviators are not exposed to the outer environment.

**Figure 3:**
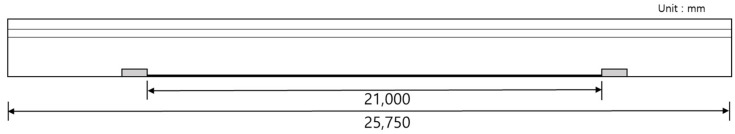
Geometry of specimen reinforced with the near-surface mounted (NSM) method.

Figure 3 shows the specimen configuration employed to study the NSM method. The total length is 25,750 mm, and the reinforcement length is 21,600 mm. The NSM method strengthens a structure by creating grooves with a constant depth and embedding FRP plates or FRP rods instead of attaching reinforcement on the concrete surface, as in the existing EB method. Kim et al. [18] reported that the NSM method affords a relatively higher performance improvement than the EB method owing to its superior resistance to bond failure. Moreover, they reported that damage due to fire or vehicular collision could be avoided, unlike in the case where the reinforcement is bonded on the surface, because the reinforcement is embedded in the concrete. In particular, when the negative moment region of the deck is reinforced with the NSM method, increased resistance to the physical wear caused by vehicles’ wheels can also be realized.

### 2.3. Flexural Test Setup

**Figure 4:**
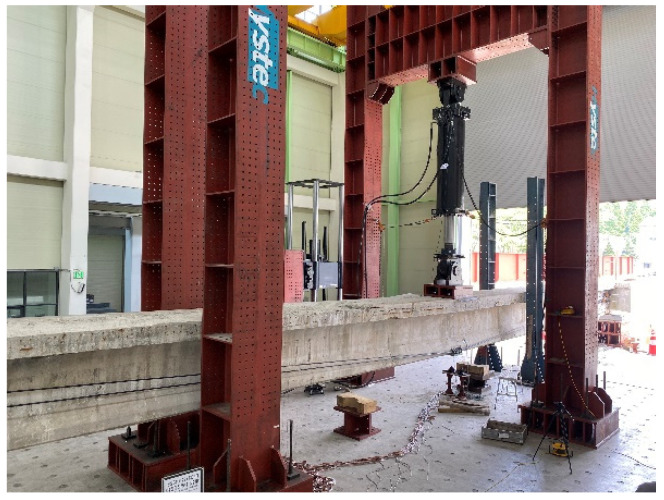
Flexural experimental setup (25 m).

**Figure 5:**
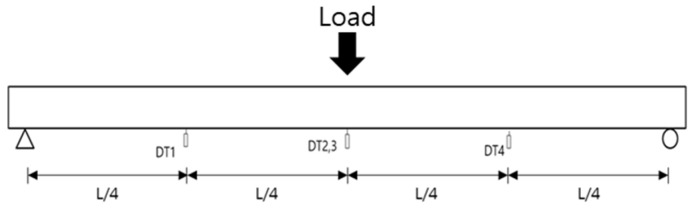
Displacement transducer (DT) positions (NSM and EP methods).

**Figure 6:**
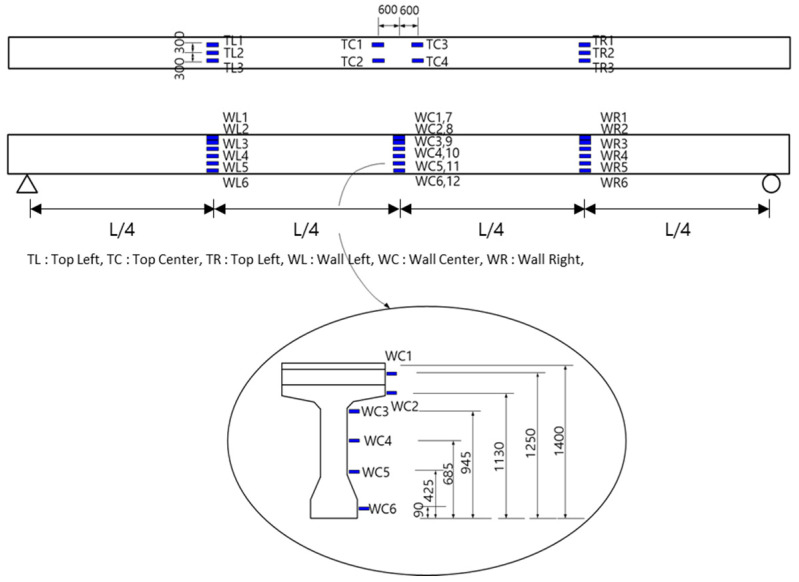
Positions of concrete gauges in the upper portion and on the side (NSM and EP methods).

**Figure 7:**
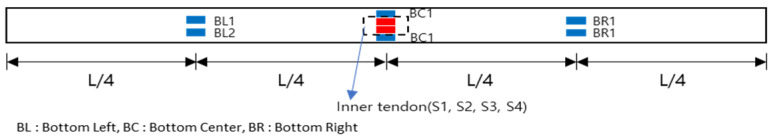
Lower concrete and inner tendon gauge positions (NSM and EP methods).

**Figure 8:**
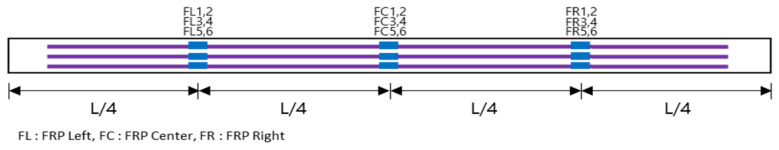
Positions of reinforcement gauges used for the NSM method.

**Figure 9:**
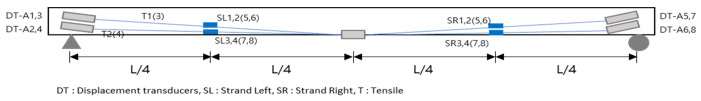
Positions of reinforcement gauges used for the EP method.

Figure 4, Figure 5, Figure 6, Figure 7, Figure 8 and Figure 9 illustrate the experimental method and the positions of the strain gauges installed on the concrete and steel. Except for the strain gauge positions on the reinforcement, the conditions of the NSM and EP methods are similar. In particular, a load was applied at the center, located at a distance of L/2 from the supports at both ends. Additionally, a 5000 kN universal testing machine (UTM, Instron_kor, Seoul, Korea) was used. Displacement transducers (DTs) (Tokyo Sokki Kenkyujo, Tokyo, Japan) were installed at a distance of L/4 from both ends. Two DTs were also placed at the center to measure the deflection according to the load. In the upper portion, three concrete strain gauges (Tokyo Sokki Kenkyujo, Tokyo, Japan) were attached at distances of L/4 from both ends, and two concrete gauges (Tokyo Sokki Kenkyujo, Tokyo, Japan) were attached at a distance of 600 mm from the center (on either side). The strain gauges were attached in this manner to avoid the loading point at the center. On the side, two concrete gauges were attached in the upper section; three concrete gauges were also installed in the middle section, while one concrete gauge was attached at the lower section.

For the lower gauge positions, two concrete gauges were attached at a distance of L/4 from both ends, and gauges were also attached at the front and rear of the groove marked at the center. This groove was created to attach strain gauges to the inner tendon, where prestressing was applied previously. For the seven-wire strand, strain gauges were attached to four wires. Figure 8 shows the positions of the reinforcement FRP gauges for the NSM method. Three gauges were attached to each reinforcement location at the L/4 points and at the center. Figure 9 shows the positions of the reinforcement gauges used for the EP method. A strain gauge was attached to the L/4 point in each of the four external strands. Additional prestressing was measured from the reinforcement used in the strengthening methods.

**Figure 10:**
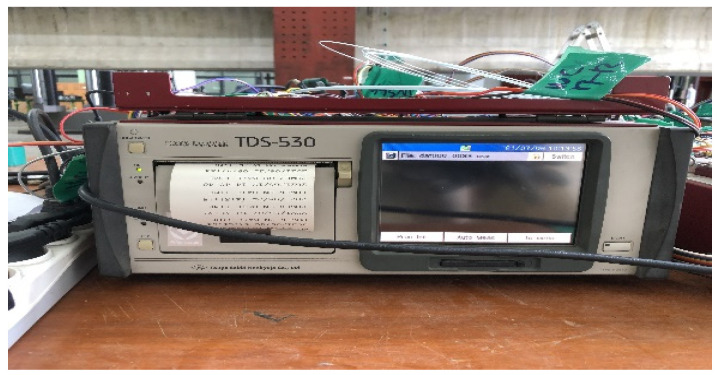
TDS-530 measuring instrument.

The measuring instrument adopted was a static electrical resistance data logger, as shown in Figure 10. In particular, the typically used TDS-530 (Tokyo Sokki Kenkyujo, Tokyo, Japan) was employed. Its built-in 30 channels were used, and the switchbox channels were used for insufficient channels.

### 2.4. Concrete Strength

The compressive and splitting tensile strengths of concrete were measured to examine the safety aspects of the deteriorated bridge. For the strength measurements, concrete cores were collected from the girder and deck of the bridge specimen, and 100 × 200 mm cylindrical specimens were prepared by cutting them and subsequently flattening the upper and lower surfaces. In addition, the tests were conducted in accordance with the KS F 2405 (Standard test method for compressive strength of concrete) and KS F 2423 (Standard test method for tensile splitting strength of concrete) standards [32,33]. Five girder and three deck specimens were tested. However, one of the deck compressive strength specimens could not be measured appropriately owing to equipment error. Table 1 and Table 2 list the compressive and splitting tensile strength results for the girder and deck. The compressive strengths of the girder and deck were 30.28 and 38.35 MPa, respectively. Their splitting tensile strengths were 3.21 and 4.96 MPa, respectively. In general, the splitting tensile strength value of concrete ranges from 1/9 to 1/13 of its compressive strength value. Thus, it was concluded that the splitting tensile strengths were measured accurately. As the strengths of the deteriorated bridge specimen were measured, the strength of the concrete immediately after completing the bridge can be expected to be higher than those shown in Table 1 and Table 2.

## 3. Flexural Test Results and Discussion

### 3.1. Additional Prestressing

#### 3.1.1. Prestressing in EP Method

Additional prestressing was performed for the EP method as follows. T1 and T3 at the top were tensioned simultaneously, considering eccentricity. Subsequently, T2 and T4 at the bottom were tensioned. Herein, prestressing was introduced in steps of 0 kN > 30 kN > 60 kN > 90 kN > 120 kN > 150 kN > 180 kN. Furthermore, strands T1 and T3 were simultaneously tensioned from 0 to 180 kN.

When T2 and T4 were tensioned, T2 was tensioned from 0 to 90 kN, followed by the tensioning of T4 from 0 to 180 kN. Finally, strand T2 was tensioned from 90 to 180 kN. Table 3 and Table 4 present the results of introducing prestressing to the strands at different steps, while Table 5 shows the final average prestressing for each strand.

When the prestressing loads of 135.17 and 144.27 kN were introduced to the left (L) and right (R) parts of T1, prestressing loads of 116.14 and 108.77 kN were introduced to the L and R parts of T3, respectively. The L–R differences were approximately 8 to 9 kN in the cases of T1 and T3, indicating that the measurements were almost identical. When prestressing loads of 134.73 and 128.43 kN were introduced to the L and R parts of T2, prestressing loads of 127.52 and 135.81 kN were introduced to the L and R parts of T4, respectively. The L–R difference was approximately 6 to 8 kN for T2 and T4, indicating that the measurements were almost identical to those for T1 and T3.

A comparison of the average values for each strand revealed that introducing the target prestressing load of 180 kN led to loads of 151.62, 136.70, 128.02, and 138.06 kN at strands T1, T2, T3, and T4, respectively. In addition, upon completing the prestressing introduction, the final prestressing levels were 139.72, 131.57, 112.45, and 131.65 kN, respectively. The final prestressing values were 92%, 96%, 87%, and 95% of those obtained when the target prestressing was introduced. Finally, the total prestressing was 515 kN, approximately 70% of the targeted 720 kN (indicating a loss of 30%).

Table 6 shows the vertical displacements when prestressing was applied to the strands. Vertical displacements of 4.3 and 5.4 mm occurred on the L and R sides of the specimen, whereas a vertical displacement of 6.05 mm occurred at the center. These results confirm that the camber phenomenon (i.e., the rising phenomenon at the center of the specimen due to prestressing) occurred when introducing prestressing. Table 7 shows the displacement of the anchorage when prestressing was introduced to the strands. Although the maximum displacement of 0.084 mm occurred at DT-A2, almost no change was observed at the other positions.

#### 3.1.2. Prestressing in NSM Method

Table 8 and Table 9 show the maximum load cell values and final strain subjected to FRP prestressing. In the case of FRP prestressing for the NSM method, prestressing was introduced in the order of L > R > center. Consequently, the final prestressing results were 87.5, 77.3, and 74.2 kN, respectively. In addition, the total prestressing was 239 kN, thus confirming almost no loss compared with the targeted 240 kN. Moreover, the final strain measurements were similar for most of the gauges. The prestressing case showed that the NSM method, which employs the screw tap, exhibited a smaller prestressing loss, compared to the EP method, which adopts the wedge type.

### 3.2. Load–Displacement

#### 3.2.1. Analysis before Applying EP Method

**Figure 11:**
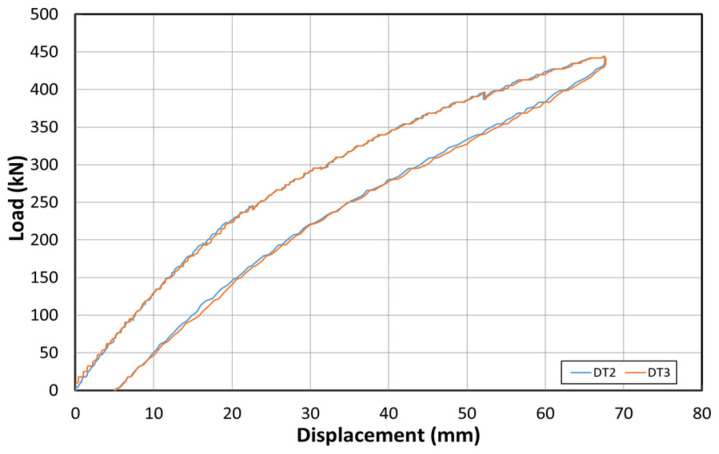
Load–displacement curve before reinforcement based on the EP method.

**Figure 12:**
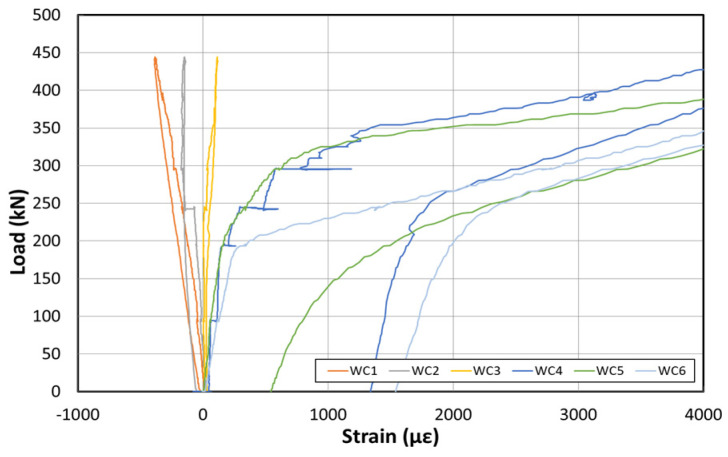
Concrete strain curve at the center before reinforcement based on the EP method.

**Figure 13:**
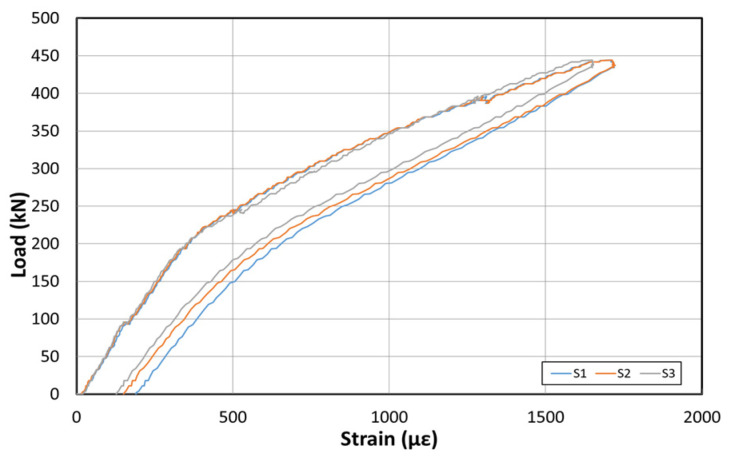
Inner tendon strain curve at the bottom before reinforcement based on the EP method.

Figure 11 shows the load–displacement graph obtained by applying the load first to examine the stiffness of the structure, before reinforcement using the EP method. Figure 12 shows the concrete strain (×10^−6^) as a function of the load. Moreover, Figure 13 shows the strain of the inner tendon according to the load. The load was measured up to 800 kN after reinforcement to allow for comparisons between the strengthening methods. The displacement control method was used for load control, and loading was performed at a rate of 0.5 mm/min. For the loading tests before reinforcement, loads up to 400 kN were measured.

The load–displacement curve shows that the initial crack occurs at approximately 210 kN, and a displacement of approximately 22 mm is also noted. Moreover, a displacement of approximately 55 mm occurred when the maximum load of 400 kN was applied. The fundamental initial stiffness before cracking was approximately 9.5 kN/mm. Among the six concrete strain gauges located on the side of the center, the strain gauges in the upper section showed almost no change in the strain. However, the strain gauge at the bottom indicated strains up to 0.006. The strain under 210 kN (initial crack) was 0.002.

**Figure 14:**
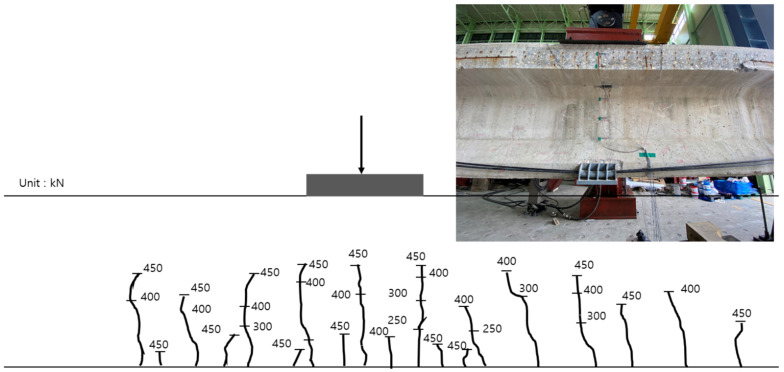
Crack map according to the load before reinforcement using the EP method.

The inner tendon showed a shape similar to that of the load–displacement curve. A strain of approximately 0.0004 was observed for loads up to 210 kN (initial crack), whereas a strain of 0.0014 was observed at the maximum load of 400 kN. Note, that no significant strain occurred in the inner tendon. Figure 14 shows the crack map. It should also be noted that the cracks are evident under loads exceeding 250 kN at the center of the girder and near the bottom and R sides of the loading point. Subsequently, the flexural tensile cracks propagated from the loading point toward both sides for loads up to 300 kN. At 400 kN, various cracks mainly occurred in the ±3 m interval from the center to both sides. In particular, some cracks even propagated to the upper portion.

#### 3.2.2. Analysis after Reinforcement via EP Method

**Figure 15:**
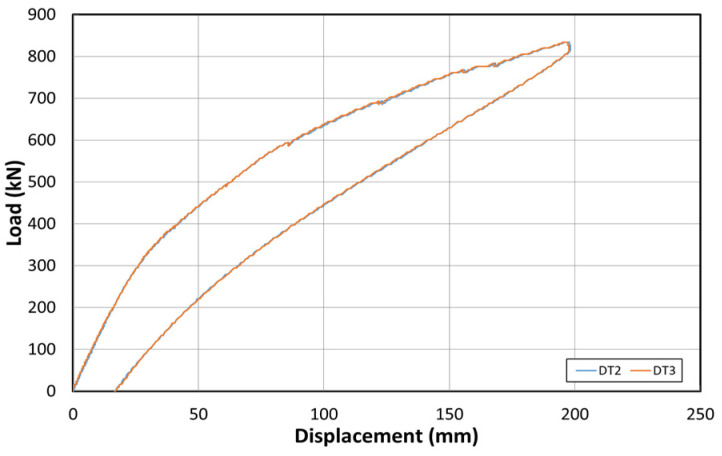
Load–displacement curve after reinforcement based on the EP method.

**Figure 16:**
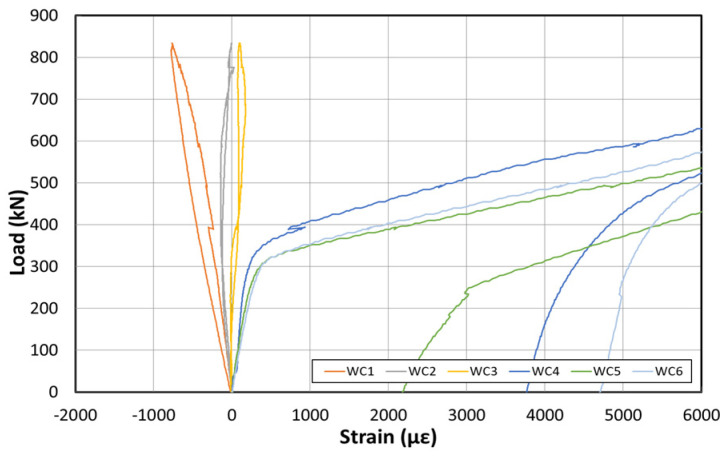
Concrete strain curve at the center after reinforcement based on the EP method.

**Figure 17:**
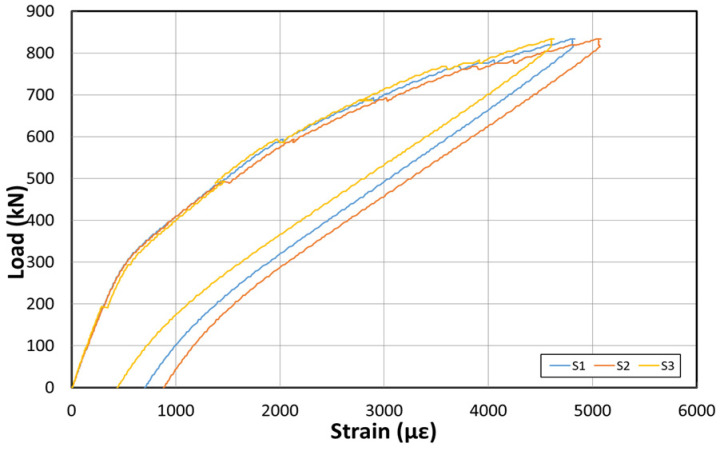
Inner tendon strain curve at the bottom after reinforcement based on the EP method.

Figure 15 shows the load–displacement graph after reinforcement based on the EP method. Figure 16 shows the concrete strain (×10^−6^) as a function of the load. Moreover, Figure 17 shows the strain response of the inner tendon as a function of the load. After reinforcement, loads up to 800 kN were measured. The displacement control method was also used for load control, similar to that before reinforcement, and loading was performed at a rate of 0.5 mm/min.

The load–displacement curve shows that the initial crack occurs at approximately 300 kN, and a displacement of approximately 23 mm is also noted. A displacement of approximately 180 mm occurred when the maximum load of 800 kN was applied. The fundamental initial stiffness before cracking was ~13.04 kN/mm. Among the six concrete strain gauges at the side of the center, the strain gauges in the upper section yielded almost no strain changes, but the strain gauge at the bottom yielded strains up to 0.012. The strain developed for loads < 300 kN (initial crack) was 0.004. The inner tendon yielded a shape similar to that of the load–displacement curve. Moreover, a strain of approximately 0.0005 was observed for loads up to 300 kN (initial crack), whereas a strain of 0.005 was noted at the maximum load of 800 kN.

**Figure 18:**
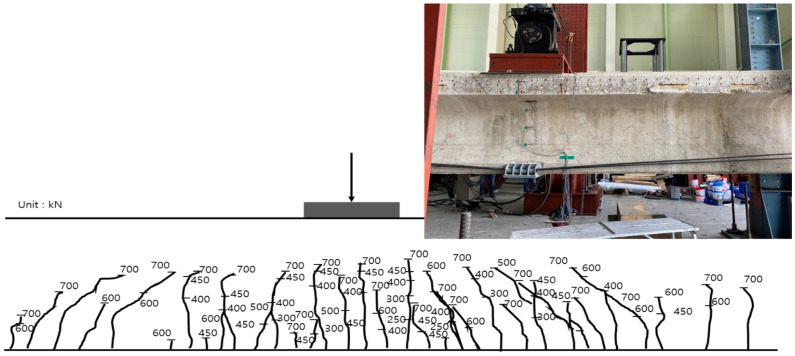
Crack map according to the load after reinforcement using the EP method.

Figure 18 shows the crack map for loads up to the maximum load. Before reinforcement, cracks mainly occurred within the ±3 m interval from the girder center to both sides. After reinforcement, as the load increased, flexural tensile cracks tended to expand to values within the ±6 m interval from the girder center on both sides.

#### 3.2.3. Analysis after Reinforcement via NSM Method

**Figure 19:**
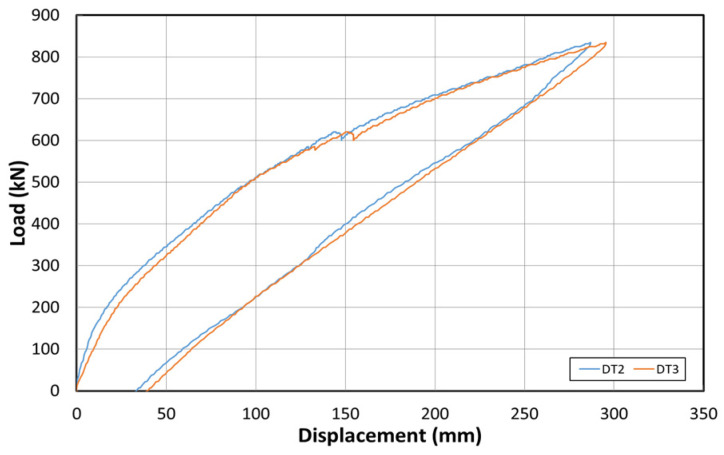
Load–displacement curves after reinforcement based on the NSM method.

**Figure 20:**
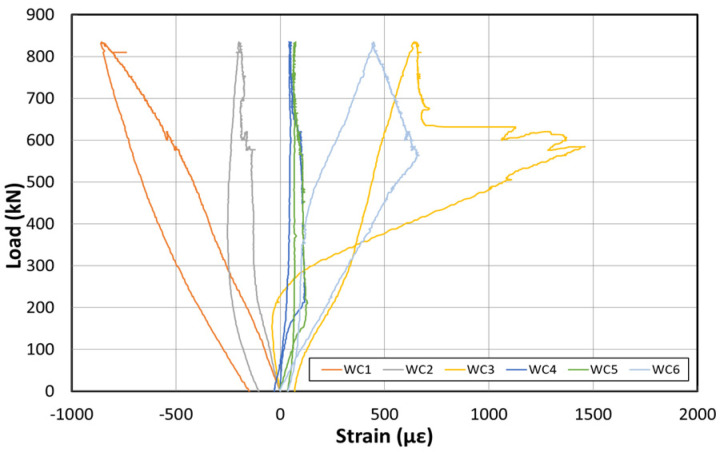
Concrete strain curves at the center after reinforcement based on the NSM method.

**Figure 21:**
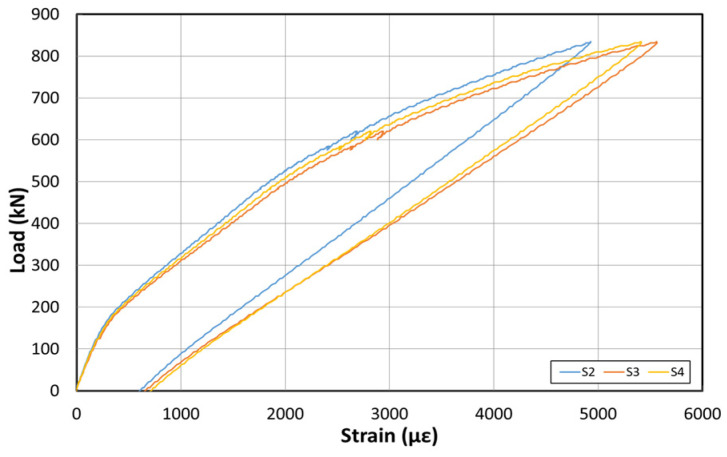
Inner tendon strain curves at the bottom after reinforcement based on the NSM method.

Figure 19 shows the load–displacement curves after reinforcement using the NSM method. Figure 20 shows the concrete strain (×10^−6^) according to the load. Moreover, Figure 21 depicts the strain of the inner tendon according to the load. After reinforcement, the load was measured up to 800 kN. The displacement control method was used for load control, as in the EP method, and loading was performed at a 0.5 mm/min rate.

The load–displacement curve shows that the initial crack occurs at ~150 kN, and a displacement of ~15 mm is obtained. At the maximum load of 800 kN, a displacement of approximately 270 mm occurred. The fundamental initial stiffness before the cracks was initiated was ~10 kN/mm. Among the concrete strain gauges on the side of the center, the strain was measured in the upper and lower sections. The maximum strain was approximately 0.008. The strain measured in the upper concrete section indicated that the failure geometry and cracks occurred in the girder of the specimen under the NSM method because the deck was significantly weak. The inner tendon exhibited a shape similar to that of the load–displacement curve. A strain of approximately 0.00025 was observed for loads up to 150 kN (initial crack), whereas a strain of approximately 0.005 was noted at the maximum load of 800 kN.

**Figure 22:**
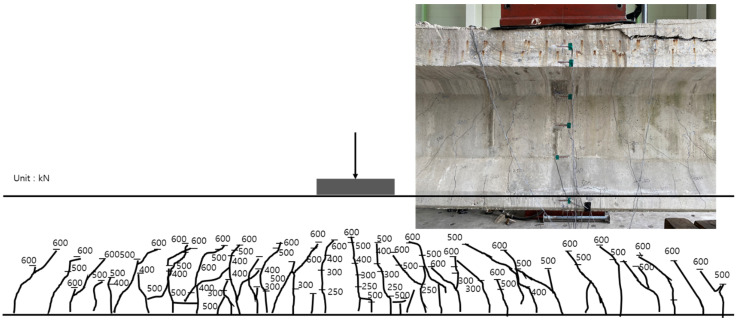
Crack map according to the load developed after reinforcement by the NSM method.

Figure 22 shows the crack map up to the maximum load after reinforcement using the NSM method. From 250 kN, cracks were evident at the center of the girder and near the bottom and right side of the loading point. Subsequently, the flexural tensile cracks propagated from the loading point to both sides for loads up to 300 kN. At 400 kN, various cracks mainly occurred within the ±3 m interval from the center to both sides. Subsequently, flexural tensile cracks propagated to the upper portion of the web and expanded to values within the ±6 m interval from the center of the girder on both sides.

### 3.3. Comparison of Strengthening Methods

**Figure 23:**
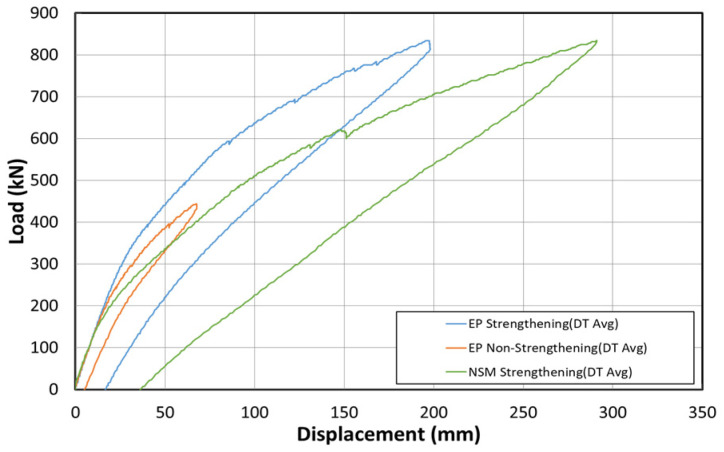
Load–displacement curves associated with the strengthening methods studied herein.

Figure 23 shows the load–displacement curves at the center after the non-strengthening, EP, and NSM methods were applied. For these methods, similar tendencies were observed for approximately 180 to 200 kN. In the 200–400 kN load range, the EP method exhibited the highest stiffness (15 kN/mm), followed by the non-strengthening (8.5 kN/mm) and the NSM methods (5.45 kN/mm). The EP method increased the stiffness by approximately two times. However, the NSM method decreased the stiffness by 0.6 times. In the 400–800 kN load range, the stiffness was 2.58 kN/mm for the EP method and 0.7 kN/mm for the NSM method. These results confirm the positive reinforcement effect of the EP method. The reinforcement effect of the NSM method, however, was insignificant.

## 4. Conclusions

This study implemented the EP and NSM methods for an actual deteriorated bridge structure using strands and FRP materials. Moreover, the characteristics of the structure before and after reinforcement were compared. The following conclusions can be drawn. The compressive strengths of the girder and deck were 30.28 and 38.35 MPa, respectively. Note, that the strengths of deteriorated bridge specimens were measured; thus, the strengths of the concrete at the time of bridge completion could be higher than the measured strengths. Following the EP reinforcement method, under the maximum load of 800 kN, a displacement of approximately 180 mm was observed. In addition, for the reinforcement afforded by the NSM method, under the maximum load of 800 kN, a displacement of approximately 270 mm occurred. In this study, the NSM and EP methods were applied using various reinforcing materials, and the structure’s performance was compared and analyzed. The displacement differed by a factor of approximately 2 at the maximum load, and the NSM method afforded increased stiffness. This can be attributed to the difference in the demolition locations and the additional prestressing, although the NSM and EP based-specimens were procured from the same deteriorated bridge. Notably, the results of this study are expected to serve as basic data for reinforcing deteriorated bridges in operation. In future research, the load prior to reinforcement will also be measured in the case of the NSM method, in order to derive more accurate results.

## Figures and Tables

**Table 1:** Compressive and splitting tensile strengths of girder.

	Girder			
Number	Compressive		Splitting Tensile	
	Load (kN)	Strength (MPa)	Load (kN)	Strength (MPa)
1	238.00	30.30	65.70	3.29
2	227.00	28.90	52.70	2.64
3	209.70	26.70	69.90	3.50
4	267.80	34.10	62.40	3.12
5	246.60	31.40	69.60	3.48
Average	237.82 ± 19.4	30.28 ± 2.5	64.06 ± 6.3	3.21 ± 0.32

**Table 2:** Compressive strength and splitting tensile strength of deck.

	Slab			
Number	Compressive		Splitting Tensile	
	Load (kN)	Strength (MPa)	Load (kN)	Strength (MPa)
1	285.10	36.30	81.80	4.09
2	317.30	40.40	108.30	5.42
3	-	-	107.10	5.36
Average	301.20 ± 16.1	38.35 ± 2.1	99.07 ± 12.2	4.96 ± 0.6

**Table 3:** Prestressing of strands T1 and T3.

Prestressing (kN)	T1-L	T1-R	T3-L	T3-R
	(kN)	(kN)	(kN)	(kN)
0	0	0	0	0
30	21.34	21.09	20.56	23.26
60	46.71	47.77	43.80	49.79
90	71.66	74.74	66.95	76.46
120	97.84	103.19	87.02	100.06
150	124.28	131.72	111.77	126.45
180	147.02	156.23	129.85	126.21
Final	135.17	144.27	116.14	108.77
Prestressing losses	44.83	35.73	63.86	71.23

**Table 4:** Prestressing of strands T2 and T4.

Prestressing (kN)	T2-L	T2-R	T4-L	T4-R
	(kN)	(kN)	(kN)	(kN)
0	0	0	0	0
30	14.99	14.76	12.32	14.04
60	40.42	39.28	39.67	43.69
90	66.22	64.36	63.67	69.19
120	89.36	87.88	87.88	94.45
150	117.18	113.60	111.16	120.12
180	138.67	134.73	132.43	143.72
Final	134.73	128.43	127.52	135.81
Prestressing losses	45.27	51.57	52.48	44.19

**Table 5:** Final prestressing of strands T1, T2, T3, and T4.

Prestressing (kN)	T1-(Avg) (kN)	T2-Avg (kN)	T3-Avg (kN)	T4-Avg (kN)
0	0	0	0	0
30	21.23	14.88	21.92	13.18
60	47.24	39.83	46.24	41.66
90	73.19	65.28	71.69	66.45
120	100.50	88.60	93.54	91.18
150	128.00	115.38	119.10	115.63
180	151.62	136.70	128.02	138.06
Final	139.72	131.57	112.45	131.65
Prestressing losses	40.28	48.43	67.55	48.35

**Table 6:** Vertical displacement when introducing prestressing to the strands (DT: displacement transducer).

DT1 (mm)	DT2 (mm)	DT3 (mm)	DT4 (mm)	L/2Avg (mm)
4.3	6.2	5.9	5.4	6.05

**Table 7:** Displacement of anchorage when introducing prestressing to the strands.

DT-A1 (mm)	DT-A2 (mm)	DT-A3 (mm)	DT-A4 (mm)	DT-A5 (mm)	DT-A6 (mm)	DT-A7 (mm)	DT-A8 (mm)
0.005	0.084	0.055	−0.014	0.018	0.005	0.05	0.005

**Table 8:** Maximum load cell values subjected to fiber-reinforced polymer (FRP) prestressing.

Load Cell-1 (kN)	Load Cell-2 (kN)	Load Cell-3 (kN)
87.5	77.3	74.2

**Table 9:** Final strain subjected to FRP prestressing.

FC1 (με)	FC2 (με)	FC5 (με)	FC6 (με)	FC3 (με)	FC4 (με)
4245	4300	4395	4440	4169	4139

## Data Availability

The data presented in this study are available upon request from the corresponding author.
